# Evidence of a Right Ear Advantage in the absence of auditory targets

**DOI:** 10.1038/s41598-018-34086-3

**Published:** 2018-10-22

**Authors:** Giulia Prete, Anita D’Anselmo, Alfredo Brancucci, Luca Tommasi

**Affiliations:** Department of Psychological, Health and Territorial Sciences, ‘G. d’Annunzio’ University of Chieti and Pescara, Chieti, Italy

## Abstract

The Right Ear Advantage effect (REA) was explored in a white noise speech illusion paradigm: binaural white noise (WN) could be presented *i)* in isolation (WN condition), *ii)* overlapped to a voice pronouncing the vowel /a/ presented in the left ear (LE condition), *iii)* overlapped to a voice pronouncing the vowel /a/ presented in the right ear (RE condition). Participants were asked to report in which ear the voice has been perceived. The voice could be female or male, and it could be presented at 4 different intensities. Participants carried out the task correctly both in LE and in RE conditions. Importantly, in the WN condition the “right ear” responses were more frequent with respect to both the chance level and the “left ear” responses. A perceptual REA was confirmed both in LE and RE conditions. Moreover, when the voice was presented at low intensities (masked by WN), it was more frequently reported in the right than in the left ear (“illusory” REA). A positive correlation emerged between perceptual and illusory REA. Potential links of the REA effects with auditory hallucinations are discussed.

## Introduction

A left-hemispheric superiority for language processing has been established since the pioneering discoveries by Broca^1^. About 90% of right-handers show a clear left-hemispheric ‘dominance’ for speech, compared to about 70% of left-handers^2,3^. Moreover, a higher percentage of right-hemispheric language lateralization has been shown in left-handers than in right-handers^4,5^. This population bias is attributable to both anatomical and functional asymmetries^6^: the *planum temporale* (PT), the posterior part of the superior temporal gyrus, is more extended in the left than in the right hemisphere, and this is more evident in right-handers (i.e.^7^). Also at a functional level, the left-hemispheric superiority for language has been widely documented (i.e.^8,9^), even if it has been recently suggested that the functional hemispheric asymmetry for speech is independent of the anatomical asymmetry of the PT^10^. With regard to the origin of the left-hemispheric superiority for language, it has been proposed that a common precursor of hemispheric asymmetry for language and handedness exists^11^, possibly at an ontogenetic level^12^. Nevertheless, recent evidence seems to suggest that at a genetic level language and handedness are unrelated^13^. A relatively recent point of view posits that atypical hemispheric asymmetry for language would be related to cognitive and psychiatric disorders, such as autism^14^, developmental coordination disorder^15^, and schizophrenia^16^. A behavioral paradigm widely used to evaluate asymmetries in auditory processing is the Dichotic Listening test (DL), consisting in the simultaneous presentation of two different auditory inputs in the two ear^17^. The asymmetry found by means of this paradigm using verbal auditory stimuli is called Right Ear Advantage (REA^18^): when asked to report which of the two stimuli is heard better, listeners are more likely to report that presented in the right ear. This bias has been also confirmed by neuroimaging studies (i.e.^19,20^) and it is attributed to the left-hemispheric superiority in language processing, as a result of an inter-hemispheric balance between left and right temporal cortices. In fact, a modulation of the REA has been shown during bilateral temporal transcranial Random Noise Stimulation (tRNS^21^) but not during unilateral tRNS or transcranial Direct Current Stimulation, applied over the temporal cortex (tDCS^21,22^).

The link between REA and functional asymmetries is also evident in clinical conditions: an atypical REA is evident in dyslexia^23^, as well as in auditory hallucinations^24–28^ and in depression^29^. In particular, auditory hallucinations (AH) seem to be related to hemispheric asymmetries for language: they constitute a symptom of psychotic disorders^30^, in particular schizophrenia, but they can be present also in the non-clinical population^31^. According to Hugdahl^26^, AH are due to a deficit in top-down control occurring in prefrontal and cingulate cortex, which should inhibit a bottom-up analysis occurring in the temporal areas. In AH, the hypothesized deficit in top-down inhibition is thought to result in hyperactivity in the temporal language areas. This hypothesis was confirmed in an fMRI study carried out by Hunter and colleagues^32^ on healthy participants: the authors found a left temporal activity during silence, with a subsequent activation of the right-hemispheric homotopic regions. They concluded that this default mode auditory network can be the neural substrate of AH. In this view, during silence the default mode auditory network would be activated spontaneously, but – in healthy participants – the top-down inhibition from the frontal areas would prevent the person to hear any auditory inputs. In AH patients the loss of the frontal top-down inhibition allows the temporal cortex to be spontaneously activated, leading as a consequence an “illusory hearing”. Concordantly, a different fMRI study^33^ revealed a left temporal activation during silent gaps in familiar songs, suggesting that these areas represent the cerebral basis of spontaneous auditory imagery.

A further evidence in this frame comes from a behavioral paradigm, in which participants were asked to imagine to hear a voice or a sound only in one ear^34^. Results confirmed an imagery REA, both when the input was a voice or a sound imagined “at” or “in” one ear, as well as when participants were asked to imagine someone saying something at one ear, or to imagine moving one ear towards the mouth of a person whispering something. Asymmetries were not found when the attention of participants was shifted toward the motor aspect of the imagined action (i.e., participants were asked to imagine a person standing in front of them and *a)* that this person approached and said something to their ear, or *b)* to say something at one ear of the imagined person). This was considered as evidence of a pure auditory imagery bias, independent of the motor content. Importantly, it revealed the presence of the REA in an imagery condition, that is to say without the physical presence of auditory stimuli. In a different paradigm^35^, participants received either anodal or cathodal tDCS during a WN speech illusion task: they were presented with 5 seconds WN bursts. In some trials, in the 3rd second a voice was presented (for 1 second) at one of four possible intensities. In each trial, participants had to respond whether they had heard a voice or not. The authors found that, during anodal/cathodal tDCS over the left posterior superior temporal gyrus, false alarms were enhanced/reduced, respectively, and they concluded that the left temporal cortex is directly involved in WN speech illusions. Moreover, different studies have shown that both schizophrenic patients and non-clinical participants with AH are more prone to WN speech illusions^36,37^, even if Pries and colleagues^38^ did not find associations between hearing a voice in WN and subclinical psychotic experiences measured by means of questionnaires. The authors concluded that positive and negative schizotypy is unrelated to WN speech illusion.

Starting from this background, the main aim of the present study was to compare the lateralization of auditory verbal perception and imagery in a WN speech illusion paradigm. In all conditions, WN was binaurally presented by means of headphones. In one third of the trials it was presented alone (WN condition); in one third of the trials a voice (pronouncing the vowel /a/) was presented in the left ear (LE condition), and in one third of the trials the same voice was presented in the right ear (RE condition). In the LE and RE conditions, the voice could be a female or male real voice presented at 4 different intensity levels. Participants were instructed that a voice would be presented in all trials, but that in some trials its volume would be very low, and thus it would be difficult to hear it clearly. Importantly, in each trial they were requested to report in which ear the voice has been perceived, in a forced choice paradigm. We expected to find a higher percentage of right ear responses, both in case of the objective presentation of the voice (perceptual REA), and in the absence of any voice (illusory REA). Finally, we also hypothesized a better performance when the voice belonged to a person the same sex as the participant: such an Own-Gender Bias (OGB) was found in voice perception, at least in male listeners^39^, similarly to the well-known OGB found in face perception^40,41^, and we tested whether it is also present in the white noise speech illusion paradigm. In order to test this hypothesis, we presented half of stimuli for each gender and tested a sample of both female and male participants. Moreover, in order to verify the relationship between REA and handedness and between REA and the proneness to experience hallucinatory percepts in the non-clinical population, correlational analyses were also carried out.

## Results

### Overall analysis: REA effect

**Figure 1:**
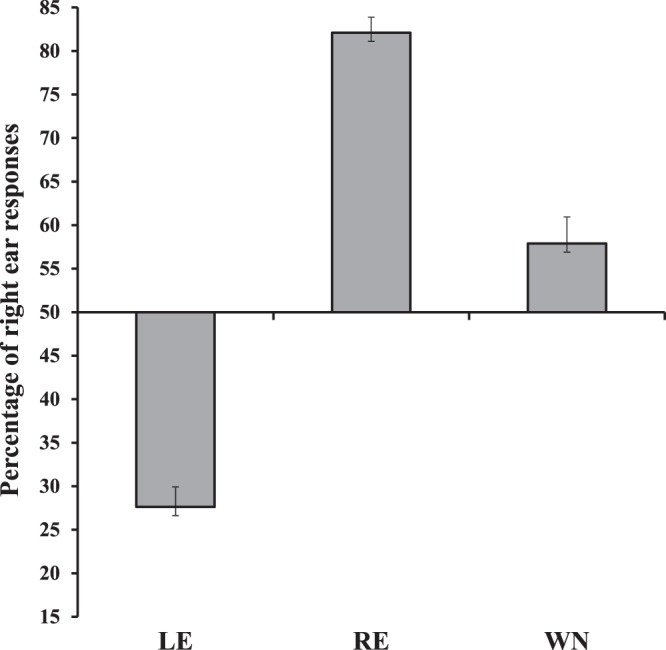
Percentage of “right ear” responses in the Left Ear (LE), the Right Ear (RE) and the White Noise (WN) conditions. Bars represent standard errors. All comparisons are significant (*p* < 0.001). Similarly, all comparisons between each condition (LE, RE, WN) and the chance level (50%) are significant.

The Kolmogorov–Smirnov normality test showed that all data were normally distributed, and the Levene test confirmed that homogeneity of variance was satisfied (raw data are available as Supplementary file). A first analysis of variance (ANOVA) was carried out by using Sex of participants (female, male) as between-subjects factor and Condition (WN, LE, RE) as within-subjects factor. The percentage of “right ear” responses was used as the dependent variable. The main effect of Condition was significant (*F*_(2,92)_ = 164.61, *MSE* = 35508.5, *p* < 0.001, *η*_*p*_^2^ = 0.78, Fig. 1), and all post-hoc comparisons, carried out by means of Duncan test, were significant (*p* < 0.001 for all comparisons). In particular, the percentage of “right ear” responses was higher in the RE condition (82.11% ± 1.78) with respect to both the LE condition (27.62% ± 2.31) and the WN condition (57.89 ± 3.05), and it was significantly lower in the LE condition than in the WN condition.

In a second analysis (Fig. 1) the percentage of “right ear” responses in each of the three conditions (WN, LE, RE) was compared with the chance level (50%) by means of exact *t*-tests. After the Bonferroni correction for multiple comparisons, the significance threshold was set at *p* = 0.017 (*p* = 0.05/3). All of the results were significant, showing that participants correctly localized the voice in the RE (*t*_(47)_ = 18.03, *p* < 0.001) and in the LE (*t*_(47)_ = −9.69, *p* < 0.001) conditions, and – importantly – revealing that when the voice was absent (WN condition), participants localized the voice more frequently in the right ear than expected by chance (*t*_(47)_ = 2.59, *p* = 0.013).

### Analysis on vocal targets

**Figure 2:**
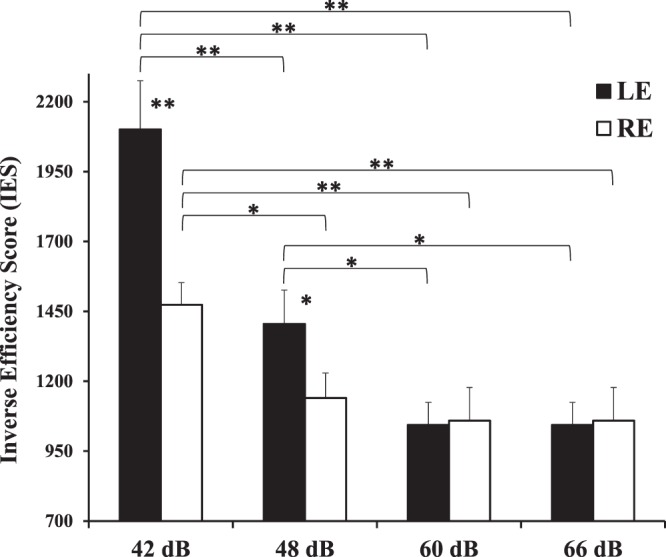
Interaction between Ear (Left Ear: black columns; Right Ear: white columns) and Intensity of the voice on the Inverse Efficiency Score. Bars represent standard errors and asterisks show significant comparisons (^*^p < 0.05; ^**^p < 0.001).

In a following analysis aimed at clarifying the laterality bias during voice presentation, only LE and RE conditions were considered. A mixed ANOVA was carried out considering Sex of participants (female, male) as between-subjects factor, and Ear of voice presentation (Left, Right), Intensity (42 dB, 48 dB, 60 dB, 66 dB), and Sex of voice (female, male) as within-subject factors. Since in this analysis the responses can be correct or incorrect (a voice was presented in all conditions), the analysis was carried out considering both accuracy and response times: the Inverse Efficiency Score (IES) was used as the dependent variable (see^41,42^), and it was obtained by dividing response times for correct responses by the proportion of correct responses in each condition. In this analysis lower scores correspond to a better and faster performance. The significant main effect of Ear (*F*_(1,44)_ = 6.86, *MSE* = 7860124.51, *p* = 0.012, *η*_*p*_^2^ = 0.13) confirmed a better performance when the voice was presented in the right ear (1167.13 ± 48.7) than in the left ear (1400.88 ± 63.28). The main effect of Intensity was significant (*F*_(3,132)_ = 24.52, *MSE* = 20678158.6, *p* < 0.001, *η*_*p*_^2^ = 0.36), and post-hoc comparisons showed that the performance was worse in the lower intensity condition with respect to all of the other conditions (42 dB: 1783.82 ± 98.18; 48 dB: 1272.34 ± 75.87; 60 dB: 1032.44 ± 58.56; 66 dB: 1051.39 ± 71.66; *p* < 0.001 for all comparisons) and it was worse when the voice was presented at 48 dB than when it was presented at both 60 dB (*p* = 0.025) and 66 dB (*p* = 0.033). The interaction between Ear and Intensity was also significant (*F*_(3,132)_ = 4.49, *MSE* = 2605668.33, *p* = 0.005, *η*_*p*_^2^ = 0.09; Fig. 2), and post-hoc tests confirmed that the performance was better in the RE than in the LE condition with intensity 42 dB (*p* < 0.001) and 48 dB (*p* = 0.039). Moreover, in the LE condition the performance was worse with intensity 42 dB than all of the other intensities (*p* < 0.001 for all comparisons), and it was worse with intensity 48 dB than 60 dB (*p* = 0.01) and 66 dB (*p* = 0.008); in the RE condition the performance was worse with intensity 42 dB than all of the other intensities (*p* < 0.003 for all comparisons).

**Figure 3:**
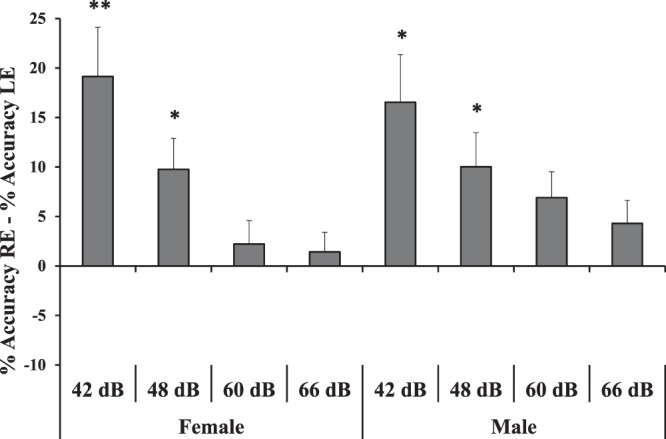
Percentage accuracy in the RE condition subtracted to the percentage accuracy in the LE condition for female and male voices presented at 42 dB, 48 dB, 60 dB, 66 dB. Bars represent standard errors and asterisks show significant comparisons when compared to 0 (absence of RE – LE difference; ^*^p < 0.05; ^**^p < 0.001).

In order to further investigate the possible laterality bias on the WN speech illusion, a series of exact *t*-tests were carried out: the percentage of the accuracy in the RE condition was subtracted to that of the LE condition for each intensity and for female and male voices separately. The resulting scores were compared against 0 (absence of right-left asymmetry). After the Bonferroni correction for multiple comparisons the threshold of significance was set at *p* = 0.006 (*p* = 0.05/8). Results were significant for both female and male voices presented at 42 dB (female: *t*_(47)_ = 3.86, *p* = 0.0003; male: *t*_(47)_ = 3.43, *p* = 0.0013) and 48 dB (female: *t*_(47)_ = 3.11, *p* = 0.0031; male: *t*_(47)_ = 2.91, *p* = 0.0056; Fig. 3).

### Correlation analysis

**Figure 4:**
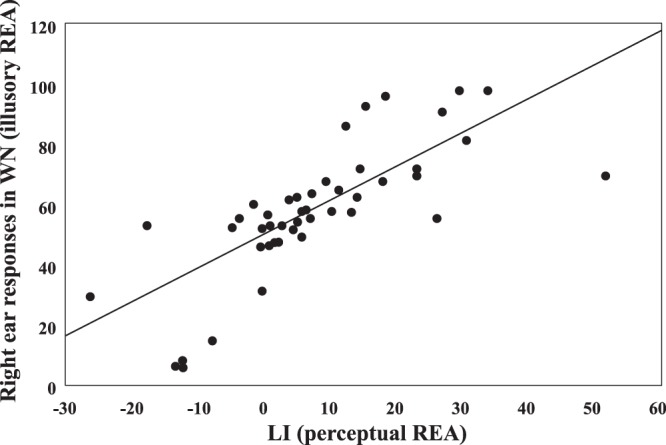
Positive correlation between right ear responses in the WN condition (illusory REA: y-axis) and LI in the LE and in the RE condition (perceptual REA: x-axis).

Finally, in order to carry out a correlation analysis, a Laterality Index (LI) was calculated by using the formula (R − L)/(R + L) × 100, where R and L correspond to the accuracy in the RE and in the LE condition, respectively. LI, in which higher scores correspond to a stronger perceptual REA, did not correlate significantly neither with the handedness score (*r* = 0.06, *p* = 0.667), nor with the unusual experiences score (*r* = 0.13, *p* = 0.357). Moreover, the handedness score and the unusual experiences score did not correlate with one another (*r* = 0.13, *p* = 0.379). A similar analysis was carried out considering the percentage of “right ear” responses in the WN condition: it did not correlate with either the handedness score (*r* = 0.01, *p* = 0.948) or the unusual experiences score (*r* = 0.16, *p* = 0.28). Finally, LI scores obtained in the conditions in which a voice was presented significantly correlated with right ear responses in the WN condition (*r* = 0.75, *p* < 0.001; Fig. 4).

## Discussion

The aim of the current study was to compare the lateralization for objective auditory perception and for subjective imagery in a white noise speech illusion paradigm. To this aim, participants were required to detect a voice within white noise in different conditions: by varying side of presentation, intensity and presence of the target stimulus. Although the target was not always present, participants were forced to report its presentation side. This paradigm, based on a controlled induction of false alarms, allowed us to test for auditory lateralization also in a condition in which the reported responses were independent of any target auditory information.

The main result of the present study indicates a preference to report right ear responses both when the vocal stimulus was present and when it was absent, thus in objective and in illusory conditions. A preference for the right ear in the absence of target acoustic stimulation had already been shown previously, in a study requiring only the mental imagery of auditory content^34^. In that study, when asked to imagine hearing a voice or a sound at only one ear, participants showed a strong preference to localize the auditory image at their right ear. The present study confirms the REA for verbal processing in the perceptual domain^18^ and, in agreement with the previous study by Prete *et al*.^34^, extends these results to the “illusory” domain. Results revealed that responses were above chance level for the right ear in the RE and for the left ear in the LE condition, indicating that the task was carried out correctly when the stimulus was objectively presented to the right or to the left ear. Furthermore, the right ear preference was higher than chance level even in the WN condition. This evidence could be explained by the structural model proposed by Kimura^17^, according to which the perceptual REA is attributable to the specific anatomical and functional organization of the human auditory pathways. In particular, the input from each ear reaches both the ipsilateral and the contralateral cortex, but – according to the model – the contralateral projections are stronger than the ipsilateral route. Thus, the auditory input from the right ear firstly and directly reaches the left-linguistic hemisphere, and this would be the structural basis for the REA. Starting from this model, we can speculate that an attentional bias extends toward the preference for the right ear to different classes of stimuli, such as white noise, as found in the present study.

Moreover, we can hypothesize that the attention of the participants was influenced by the presentation side of the stimulus and by the type of material presented (verbal stimuli). Therefore, it cannot be ruled out that the greatest number of stimuli detected by the right ear in both the LE and the RE condition might have biased in a bottom-up manner the orientation of participants’ attention toward the right ear in the illusory condition (WN), carrying over a greater number of right ear responses even in the absence of any target stimulus. This would further confirm the well documented REA effect in the perception of verbal material^43,44^. It has also to be highlighted that, differently from AVH, in the present study participants were informed that a voice was presented in every trial, so that they had to choose in which ear the stimulus was presented with WN. In this frame, it has to be noted that a voice detection test was administered to the participants before taking part to the experiment. Four participants were then excluded from the analyses because they showed a different hearing threshold between the left and the right ear. It should be noted that this pre-test was used instead of a canonical audiometric assessment. Participants were recruited only if they stated to have a normal hearing and passed the pre-test, but it is possible that some listeners had hearing loss at some frequencies, and that such loss might be asymmetrical between the two ears.

An additional aspect considered in this study is the set of different intensities the target stimulus was presented at. Importantly, the effect of voice intensity on the laterality was particularly interesting. We found that a variation in the intensity was related to a variation in the magnitude of the REA effect. The side difference was statistically significant when the volume of the target stimulus was presented at lower (42 and 48 dB) than at higher intensities (60 and 66 dB). Specifically, with lower intensities the magnitude of the REA increased. A modulation of the ear advantage when varying the intensity of the left and right ear inputs was observed also in previous dichotic listening studies^45–47^. The ability to report a syllable presented to one ear improved when that stimulus was higher in volume than the syllable presented to the other ear. Moreover the interaural intensity difference interacted with top-down attentional control in DL, showing a facilitation of the stimulus-driven laterality effect, stronger in the forced-right than in the forced-left condition when increasing the right ear intensity^47^. It should be noted that these studies differed in the methodology from our study, in which the stimuli were presented only at the left or right ear and an interaural intensity difference was not present. In any case, our findings also support an advantage of the right ear especially in a condition in which the stimulus was more difficult to detect due to the decrease of the voice intensity.

A further hypothesis was tested by presenting both female and male voices: we expected a possible preference for voices belonging to the same gender as the participants, but this OGB was not confirmed. We can conclude that the difficulty in clearly hearing vocal stimuli hidden in WN could be the reason for this null result.

Finally, the present study shows that asymmetry in the perceptual REA did not correlate with the score of participants in the unusual experiences subscale of the O-LIFE questionnaire. These results are in agreement with a recent study that did not find a correlation between speech illusion perception and schizotypy scores in a non-clinical population^38^. However, previous studies showed that participants with higher scores in the unusual experiences or the positive schizotypy subscales were more likely to perceive speech illusion in a white noise task^48,49^. Hence our results do not allow us to speculate that a different asymmetry could be predictive of a disposition to perceive unusual experiences, such as auditory hallucinations. Nevertheless, it cannot be ruled out that other psychological aspects may have produced the present lateral bias; for example in a speech detection task within white noise, trait anxiety was found to predict the likelihood of reporting more false positives under distress^50^. Other constructs should thus be investigated to study their correlation with imagery and to evaluate whether there is a relation with hemispherical lateralization.

## Conclusions

The present study extends the investigation of auditory cerebral asymmetry to the imagery/illusory domain, demonstrating a strong correspondence with the asymmetry observed in objective auditory perception. The imagery test could be a useful tool to assess lateralization also in the clinical field, for example in patients with verbal hallucinations, in which an altered perceptual REA could be related with an alteration in the illusory REA. Further studies with clinical and non-clinical participants could focus on other psychological aspects potentially correlated to the illusory REA. Moreover a similar test could be useful in order to test in advance the development of pathologies in the perceptual domain, such as auditory hallucinations. Importantly, the present study shows that a REA is also evident in a WN speech illusion paradigm, stressing the importance of attentional bias in this frame, going beyond the pure perceptual domain, to the imagery and illusory domain.

## Materials and Method

### Participants

A sample of 52 right-handed participants (26 females) took part in the study. The mean age of the sample was 24.31 years (±0.82 years) and the mean handedness score was 71.51 (±2.16), as measured by means of the Edinburgh Handedness Inventory^51^, in which a score of −100 corresponds to a complete left preference and a score of +100 corresponds to a complete right preference. Participants also completed the short version of the Unusual Experiences scale of the Oxford-Liverpool Inventory of Feelings and Experiences questionnaire (O-LIFE^52^), composed of 12 items about perceptual aberrations, magical thinking, and hallucinations. The score of the scale is comprised between 0 and 12, with higher scores being related to the positive symptoms of psychosis. The scores of the sample were comprised between 0 and 9, with a mean of 4.67 (±0.33).

Participants gave their informed consent to take part into the study as volunteers, and they were enrolled if they did not report any auditory impairment. An audiometric assessment was performed prior to the beginning of the task, in which participants had to press a button when a voice presented via earphones became perceivable to each ear. In particular, a female voice pronouncing the letter /a/ was presented repeatedly in each ear, with increased intensities (2 dB steps). Participants were recruited when no different hearing thresholds were present between left and right ear (10 dB): for this reason, 4 participants (3 females) had to be excluded from the analyses. All participants declared to be free from psychiatric or neurological disorders. The study conformed to the principles expressed in the Declaration of Helsinki and was approved by the Comitato etico per la ricerca “G. d’Annunzio”.

### Stimuli

White noise (250 ms duration) was created by using GoldWave v5.25 software (GoldWave Inc., Canada). Four female and four male voices were recorded and vocal stimuli were created by using only the phoneme /a/ lasting 250 ms. Both WN and vocal stimuli were modified by inserting a linear fade in lasting 50 ms and a linear fade out lasting 70 ms, at 60 dB. Female and male voices were then transformed in order to obtain three different intensities with respect to the original voice (60 dB), one higher than the original one, namely at 66 dB, and two lower than the original one, namely 42 dB and 48 dB.

WN stimuli were constituted of white noise presented at 60 dB binaurally. The other stimuli were instead created by superimposing to the binaural WN (60 dB) each of the 4 female and 4 male voices (8 stimuli) at each of the 4 intensities (42 dB, 48 dB, 60 dB, 66 dB) in the left and in the right channel, separately.

### Procedure

Participants were tested in isolation in a quiet room, in which no external auditory inputs could be heard. They were asked to wear headphones, to seat in front of a computer screen and to gaze at a fixation cross presented in the center of the screen for the whole duration of the experiment. In a trial, after 500 ms in which only the cross was present, an auditory stimulus was delivered (duration: 250 ms). Participants were instructed to localize the voice as perceived in the left ear or in the right ear, and to report it by pressing two different keys, after which the following trial started. Participants were informed that the voice could be female or male, that it would be presented together with a noise, and that it could be presented at different volumes so that in some trials it would have been difficult to distinctively hear it. They were asked to press the key “z” with the left hand if they believed the voice had been presented to the left ear, and the key “m” with the right hand if they believed it had been presented to the right ear. They were also asked to be fast and to give the first response they believed to be correct.

The task was controlled by means of E-Prime software (Psychology Software Tools, Inc., Pittsburgh, PA) and it was composed of 96 stimuli, repeated 4 times, for a total of 384 trials. Among the original 96 stimuli, 32 were WN presented in both ears without any superimposed voice (WN condition), 32 were binaural WN superimposed to a voice presented in the Left Ear (LE condition), and 32 were binaural WN superimposed to a voice presented in the Right Ear (RE condition). In both LE and RE conditions, 16 stimuli contained a female voice and 16 contained a male voice. In each subgroup, the four female and male voices were presented at each of the four intensities (42 dB, 48 dB, 60 dB, 66 dB). The order of stimuli was randomized within and among participants.

Prior to the beginning of the task, four trials were presented in order for the participants to familiarize with the paradigm. After the end of the task, participants were invited to complete the Unusual Experiences scale and the Edinburg Handedness Inventory, and then they were debriefed.

## Electronic supplementary material

Dataset 1
